# Supplementary material for: Gender disparities in application and admission to the medical residency program in Peru: A cross sectional study from 2016 to 2023
Source: PLoS One. 2025 Jan 3;20(1):e0316859. doi: 10.1371/journal.pone.0316859 (PMC11698358; doi:10.1371/journal.pone.0316859)
Supplement: Attachment — Submitted filename: V2 Response to reviewers.docx [file pone.0316859.s002.docx]

**Manuscript ID: PONE-D-24-38457R1**

**Title: “Gender disparities in the medical residency program in Peru: an analysis of applications and admissions from 2016 to 2023”**

| **Journal Requirements:** | **N°** | **Comments** | **Localization** | **Response** |
| --- | --- | --- | --- | --- |
| #1 | 1 | Please review your reference list to ensure that it is complete and correct. If you have cited papers that have been retracted, please include the rationale for doing so in the manuscript text, or remove these references and replace them with relevant current references. Any changes to the reference list should be mentioned in the rebuttal letter that accompanies your revised manuscript. If you need to cite a retracted article, indicate the article’s retracted status in the References list and also include a citation and full reference for the retraction notice | General | We appreciate your comment.  We have thoroughly reviewed the reference list and can confirm that no retracted papers were found. All cited references are current and accurate. |

| **Additional Editor Comments** | **N°** | **Comments** | **Localization** | **Response** |
| --- | --- | --- | --- | --- |
| #1 | 1 | The authors have satisfactorily restructured their manuscript, broadened their discussion and clarified methodological strategy. Some proof reading and grammatical correction would significantly improve the quality and readability of the manuscript. | General | We appreciate your comment.  We have carefully proofread the manuscript and made several grammatical corrections to enhance the quality and readability. |
